# Gross primary productivity and water use efficiency are increasing in a high rainfall tropical savanna

**DOI:** 10.1111/gcb.16012

**Published:** 2021-12-23

**Authors:** Lindsay B. Hutley, Jason Beringer, Simone Fatichi, Stanislaus J. Schymanski, Matthew Northwood

**Affiliations:** ^1^ Research Institute for the Environment and Livelihoods, College of Engineering, IT & Environment Charles Darwin University Casuarina Northern Territory Australia; ^2^ School of Agriculture and Environment The University of Western Australia Crawley Western Australia Australia; ^3^ Department of Civil and Environmental Engineering National University of Singapore Singapore Singapore; ^4^ Environmental Research and Innovation Department, Catchment and Eco‐hydrology Group (CAT) Luxembourg Institute of Science and Technology Belvaux Luxembourg

**Keywords:** CO_2_ fertilization, ecosystem model, eddy covariance, Howard Springs, water use efficiency

## Abstract

Despite their size and contribution to the global carbon cycle, we have limited understanding of tropical savannas and their current trajectory with climate change and anthropogenic pressures. Here we examined interannual variability and externally forced long‐term changes in carbon and water exchange from a high rainfall savanna site in the seasonal tropics of north Australia. We used an 18‐year flux data time series (2001–2019) to detect trends and drivers of fluxes of carbon and water. Significant positive trends in gross primary productivity (GPP, 15.4 g C m^2^ year^−2^), ecosystem respiration (*R*_eco_, 8.0 g C m^2^ year^−2^), net ecosystem productivity (NEE, 7.4 g C m^2^ year^−2^) and ecosystem water use efficiency (WUE, 0.0077 g C kg H_2_O^−1^ year^−1^) were computed. There was a weaker, non‐significant trend in latent energy exchange (LE, 0.34 W m^−2^ year^−1^). Rainfall from a nearby site increased statistically over a 45‐year period during the observation period. To examine the dominant drivers of changes in GPP and WUE, we used a random forest approach and a terrestrial biosphere model to conduct an attribution experiment. Radiant energy was the dominant driver of wet season fluxes, whereas soil water content dominated dry season fluxes. The model attribution suggested that [CO_2_], precipitation and *T*_air_ accounting for 90% of the modelled trend in GPP and WUE. Positive trends in fluxes were largest in the dry season implying tree components were a larger contributor than the grassy understorey. Fluxes and environmental drivers were not significant during the wet season, the period when grasses are active. The site is potentially still recovering from a cyclone 45 years ago and regrowth from this event may also be contributing to the observed trends in sequestration, highlighting the need to understand fluxes and their drivers from sub‐diurnal to decadal scales.

## Graphical abstract

Long‐term eddy covariance measures over an 18‐year period show positive trends in carbon fluxes, water use and radiation efficiencies (WUE, RUE) as well as an index of growing season length (GS). The climate and phenology of the region are strongly seasonal and variables with significant trends in each season are shown. Trends in fluxes were largest in the dry season, implying that woody components were a larger contributor to the trends in fluxes than the grassy understorey. The trends were also reproduced using a mechanistic model and a simple attribution experiment suggested CO_2_ fertilization and a weak wetting trend were the likely drivers of the observed trends.

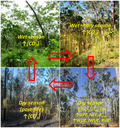

## 1 INTRODUCTION

The increasing length of the satellite record, long‐term (>50 years) monitoring of atmospheric CO_2_ concentration and the expansion of a global eddy covariance (EC) monitoring network (Baldocchi, 2020; Baldocchi et al., 2018) now enable a greater focus on interannual variability and temporal trends in the carbon cycle and its drivers (Chen et al., 2019; Fu et al., 2019; Jung et al., 2017; Von Buttlar et al., 2018; Zscheischler et al., 2016). The increase in the global terrestrial carbon sink is well documented (Anav et al., 2015; Ciais et al., 2019; Le Quéré et al., 2018; Raupach et al., 2008; Schimel et al., 2015; Sitch et al., 2015) and there is evidence from leaf to global scale modelling and observation that CO_2_ fertilization could be a major driver of the currently observed land C sink (Campbell et al., 2017; Devaraju et al., 2016; Sitch et al., 2015; Sun et al., 2018; Walker et al., 2021; Yakir, 2017). Tropical ecosystems are thought to be responsible for much of this sink (Schimel et al., 2015; Zhu et al., 2016), although there is a degree of uncertainty related to the magnitude and trend of this sink (de Meira Junior et al., 2020; Fu et al., 2017; Gatti et al., 2014; Gloor et al., 2012), especially in seasonal wet–dry tropics (Cleverly et al., 2016; Poulter et al., 2014). Identifying the regions responsible for uptake and/or loss of carbon remains an important constraint for improving estimates of the global carbon balance (Ballantyne et al., 2012). Given the spatial extent of tropical savanna (27.6 Mkm^2^, Hutley & Setterfield, 2019), quantifying savanna carbon dynamics will contribute to reducing the uncertainty associated with the tropical land sink.

Savannas are defined by the coexistence of both woody (trees, shrubs) and grass lifeforms and are transitional between grasslands and forests (Scholes & Archer, 1997; Torello‐Raventos et al., 2013). Savannas together with tropical forests are responsible for ~60% of global gross primary productivity (GPP; Beer et al., 2010). Savanna climates feature alternating wet and dry seasons that result in ‘boom‐bust’ cycles of growth followed by dry, fire‐prone periods. Given the spatial extent and high productivity of tropical forests and savanna (Beer et al., 2010), these two biomes dominate land‐atmospheric carbon feedbacks and interannual variability of atmospheric CO_2_ concentration (Cox et al., 2013; Poulter et al., 2014). Climate change is likely to have significant effects on savanna ecosystems in terms of structure (tree–grass ratio), function (productivity, water use efficiency [WUE] and light use efficiency) and biodiversity (Midgley & Bond, 2015). Savanna productivity is largely driven by rainfall variability, particularly in ‘pulse‐driven’ semi‐arid savannas (Archibald et al., 2009; Cleverly et al., 2013; Ma, Baldocchi, et al., 2016; Ma, Huete, et al., 2016) and increased interannual variability of climatic drivers, as is currently being observed in savannas, grasslands and arid lands (Chen et al., 2019), may result in rapid swings in productivity and shifts in fire climatology and thus fire regimes.

These processes are difficult to capture using terrestrial ecosystem models, especially the simulation of fire impacts and resprouting capability of savanna vegetation (Scheiter et al., 2013; Whitley et al., 2017). Further complexity arises from the differential growth responses of C_3_ trees and C_4_ tropical grasses to temperature, CO_2_, drought and N fertilization (Midgley & Bond, 2015; Moncrieff et al., 2016; Scheiter et al., 2015). Clearly quantifying mean values and trends in GPP, net ecosystem exchange (NEE) and ecosystem respiration (*R*_eco_) from savannas will lead to a better understanding of their sensitivity to drivers such as atmospheric CO_2_ concentration, precipitation, radiant energy and temperature.

Such data are being provided by the global EC flux network (FLUXNET) that has expanded over the last 25 years to ~600 sites (Baldocchi et al., 2018; Jung et al., 2020), where GPP, NEE and *R*_eco_ are being quantified across the earth's major biomes. However, tropical savannas are at present poorly represented across this network (Baldocchi et al., 2018), with few long‐term sites located in savanna ecosystems. The EC method provides data over a range of temporal scales that enable insights into the short‐term (hourly) changes in biophysical drivers of fluxes and effects of short‐term ‘pulse’ events (heat waves, fire events, storms), as well as decadal trends in ecosystem responses to climate variability and land use change. This is particularly important in savanna environments given the high interannual variability of rainfall and frequent disturbance from events such as fire (Archibald et al., 2009; Chen et al., 2019; Ma, Huete, et al., 2016; Ma et al., 2020), storm damage and cyclones (Hutley et al., 2013; Williams & Douglas, 1995). Modelling of NEE seldom incorporates disturbance factors or forest age and physiological processes can respond differently to environmental drivers following disturbance (Besnard et al., 2018; Scheiter et al., 2013).

Here we report mean values and trends in fluxes of carbon (GPP, NEE and *R*_eco_) and water (latent energy [LE]) and examine the relative importance of biophysical drivers at a tropical savanna site in north Australia, the Howard Springs EC site. We investigated the behaviour of WUE and radiation use efficiency (RUE) using an 18‐year time series (2001–2019) of local meteorological and flux data to (1) detect significant trends in site meteorology, GPP NEE, *R*_eco_ and LE at annual and seasonal temporal scales; (2) quantify trends in WUE and RUE and (3) determine the relative importance of drivers and identify physiological processes underpinning any observed trends in fluxes using a random forest analysis and mechanistic ecohydrological model (Tethys‐Chloris [T&C] model, Fatichi et al., 2012) in an attribution experiment. This approach provides a fine‐scale, bottom‐up assessment of trends and drivers of carbon and water exchange from a tropical savanna, an ecosystem that dominates the wet–dry tropics.

## 2 MATERIALS AND METHODS

### 2.1 Study site

The Howard Springs EC site (Fluxnet site code, AU‐How) is located within a gazetted water management area of the Howard River catchment (126 km^2^) located 35 km south‐east of Darwin, Northern Territory, Australia. The site was established in the mid‐1990s, with initial work focussing on groundwater dependence and water balance of the savanna vegetation via short‐term EC measurement campaigns (Cook et al., 1998; Eamus et al., 2001; Hutley et al., 2000). Since August 2001, flux observations have been made continuously (Beringer et al., 2007, 2016; Moore et al., 2017, 2018). The site is subjected to frequent fire (two in every 3 years) and storm events with significant interannual variability in rainfall (Hutley & Beringer, 2011). The site is located ~25 km from the coast and in a region subject to tropical cyclones (Cook & Nicholls, 2009) with two significant events affecting the region over the last century.

The vegetation of the catchment is a mosaic of Eucalypt‐dominated open‐forest and woodland savanna, seasonally flooded swamps and wetlands. Open‐forest and woodland savanna is the dominant vegetation type, occupying ~75% of the catchment, and flux measurements were made in this vegetation type (Duvert et al., 2020). Savanna vegetation at the site consists of three strata: an overstorey dominated in terms of leaf area and biomass by two Eucalypt species, *Eucalyptus tetrodonta* (F. Muell.) and *E*. *miniata* (Cunn. Ex Schauer) of mean canopy height of 18 m; a mid‐storey of semi‐deciduous to fully deciduous trees and shrub species between 2 and 8 m; and an understorey of tall C4 grasses dominated by *Sorghum intrans*, *S*. *plumosa* and *Heteropogon triticeus* and woody saplings (Hutley et al., 2011; Moore et al., 2016). *E*. *tetrodonta*–*E*. *miniata*‐dominated tall‐grass savanna types are widespread and dominate the Darwin Coastal bioregion that occupies 27,800 km^2^ of the north‐western coastline of the Northern Territory. The seasonal climate drives a highly dynamic phenology, with leaf area index (LAI) of the woody canopy typically ranging from 0.6 to 1 over the dry to wet seasonal cycle (Moore et al., 2016; O'Grady et al., 2000). The seasonality in cover is largely driven by the seasonal phenology of non‐Eucalypt tree and shrub species that dominate the mid‐ and upper‐canopy (Williams et al., 1997). The green LAI of the grass‐dominated understorey is more dynamic and ranges from near zero in the late dry season (August–September) to a maximum of 1.5 prior to seed set during the peak of the wet season in March. Following seed set of the annual grass, understorey LAI rapidly declines from late March.

This savanna type occurs on highly weathered Cainozoic sand deposits that form extensive, uniform, gently undulating plains (Fox et al., 2001) of low nutrient, red and grey Kandosol soils (Isbell, 2002). Soils at the site are freely draining red Kandosols with a sandy‐loam texture (sand fraction 77%–81% at 0.1 and 1.0 m depths, respectively) and a high gravel content (20%–50% by volume). At 1.5–2 m depth, ferricrete boulders occur in a matrix of mottled heavy clays forming a duricrust of low permeability and variable depth. Prominent macropores, often containing tree roots or old root channels, are found in this layer (Hutley et al., 2000). Soil organic carbon ranges from 2.2% to 0.23% from 0.1 to 1.0 m depths with a soil C:N ratio of 33.1 to 13.6, respectively (Livesley et al., 2011).

### 2.2 Fire regime and management

North Australian savanna is a highly flammable landscape with fire frequency typically increasing with mean annual precipitation (MAP) (Russell‐Smith & Yates, 2007). Fire districts around Darwin have one of the highest frequencies and largest burnt areas in the NT due to high rainfall that drives fuel production (Bowman et al., 2007), and a high population density. The Howard River catchment has limited fire management and some fraction of the catchment is burnt every dry season (April–October), largely caused by human ignition. Early dry season fires (late April–May) are typically of low intensity (500–2000 kW m^−1^), whereas late dry season fires (August–September) have intensities of 5000–7000 kW m^−1^ when fuel loads have accumulated and are fully cured (Williams et al., 1998). The flux footprint (~1 km^2^) was subjected to fires in 13 of the 18 years of the observation period, with events of varying severity and a fire line intensity ranging from 900 to 3600 kW m^−1^ (Beringer et al., 2007). These intensities are relatively low and crown fires do not occur, and continuous flux measurements are possible at the site as protective fire breaks were installed around the flux tower infrastructure. Fire fronts move through the fetch area, but, due to the fire breaks, do not affect the instrument performance, except in August 2009, when instrument systems were damaged. Fire events in the vicinity of the tower violate assumptions of the EC technique, especially stationarity of fluxes. Data were gap filled (described below) and for fire events, were excluded for a period of 60 days following fire events as quantified by Beringer et al. (2007) who examined the flux dynamics immediately following fire.

### 2.3 Flux and microclimate measurements

**FIGURE 1:**
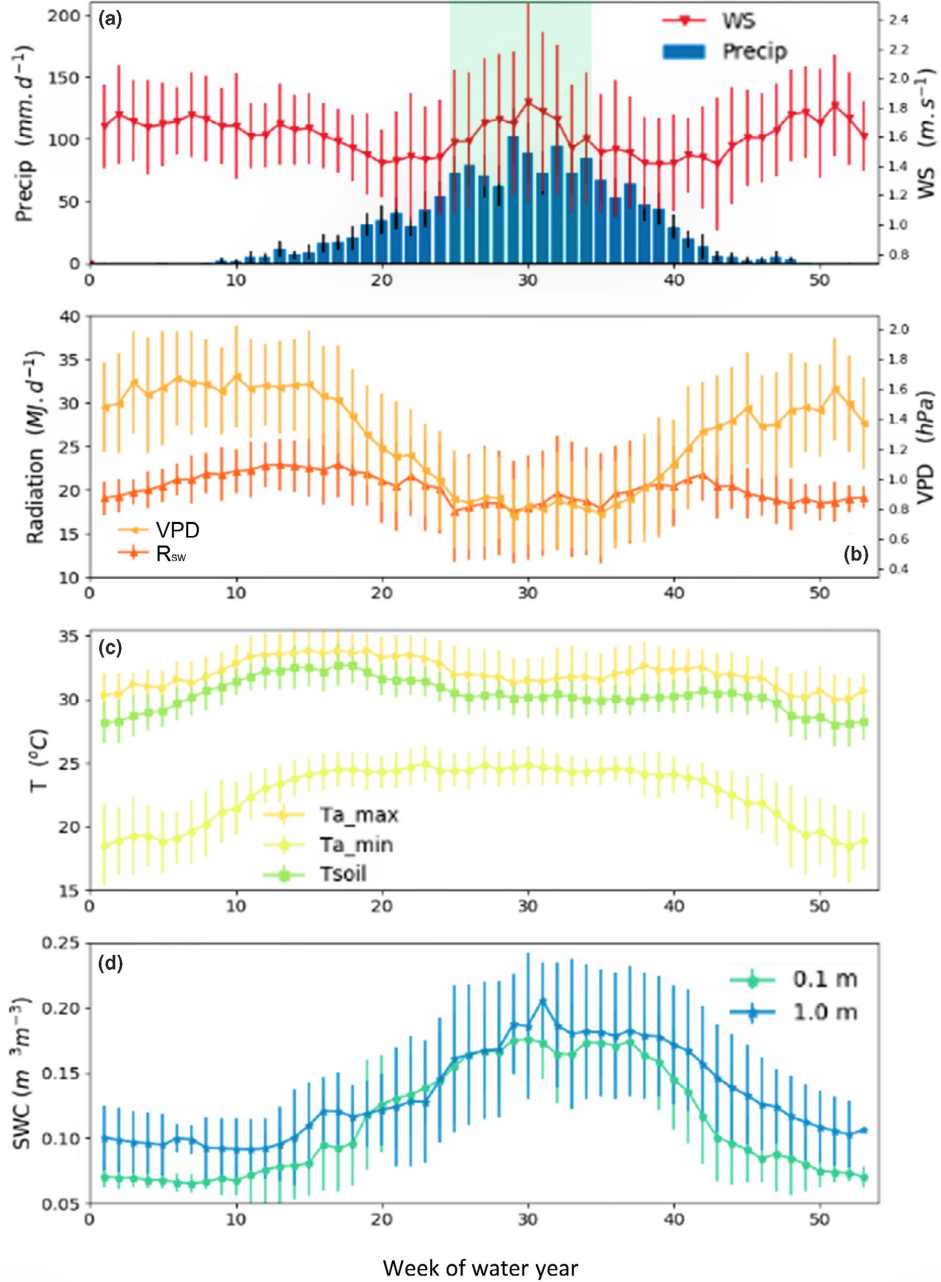
Ensemble weekly means ± standard deviation (SD) for (a) rainfall (Precip), wind speed (WS), (b) downwelling shortwave radiation (*R*_sw_), site vapour pressure deficit (VPD), (c) maximum, minimum air temperature (*T*_max_, *T*_min_) and soil temperature (*T*_s_) and (d) volumetric water content (*θ*_v_) at 0.1 and 1 m depths. Mean values were calculated by grouping data by week of the year across all water years (1 July to 30 June). Blue shading represents the peak wet season weeks

We measured half‐hourly fluxes of heat (*H*), water (LE) and net CO_2_ flux (NEE) over an 18‐year period from August 2001 to August 2019. Mean canopy height was 18 m and flux instruments were mounted on a 23 m guyed mast with adequate homogeneous fetch in all directions (~0.7 to 1.5 km, slopes <1°). Core instrumentation consisted of a 3D ultrasonic anemometer (model CSAT3; Campbell Scientific, Inc.) and an LI‐7500 open‐path CO_2_/water vapour analyser (Licor, Inc.). Flux variables were sampled at 10 Hz, with 30 min mean covariances calculated and stored. Air temperature (*T*_air_) and absolute humidity were measured at the instrument height. Soil temperature (*T*_soil_) was measured using an averaging thermocouple over the top 8 cm of soil in combination with soil heat plates to estimate soil heat flux (*G*). Volumetric soil water (*θ*_v_) was measured at 10, 40, 100, 120 and 140 cm depths using CS616 TDR probes (Campbell Scientific). All soil sensors were located within a 10 m radius of the tower base (Beringer et al., 2003). We used the seasonal dynamics of *θ*_v_ at 10 cm depth as a proxy for growing season length (GS), defined as the number of days of each water year when *θ*_v_ at 10 cm depth was >0.1 m^3^ m^−3^. This soil moisture threshold is typically observed during transition and wet season periods, that is, mid‐October to early May (Figure 1d). In effect, this is the number of ‘wet days’ per year; wet days are not necessarily consecutive, but will only occur during this period of the year when soil water is non‐limiting as this *θ*_v_ corresponds to a matric potential of ~0.03 MPa at 10 cm depth in these sandy‐loam soils (Calder & Day, 1982; Kelley, 2002). The number of days that meet this criterion can be calculated on a weekly, seasonal or annual basis using data from our 18‐year observational period.

Air temperature data were used to compute a daily mean *T*_air_ and daily maximum and minimum temperatures (*T*_max_, *T*_min_). The *T*_air_ data were combined with mean daily RH% to compute vapour pressure deficit (VPD). Surface radiant energy balance was measured using a CNR1 and CNR4 all‐wave radiometer (Kipp and Zonen) that consists of downwelling and upwelling shortwave and longwave radiation. Net radiation (*R*_n_) was also measured independently using an integrated net radiometer (Q7; Radiation and Energy Balance Systems, Inc., Seattle and an NR‐Lite; Kipp and Zonen). The gas analyser was stable and was recalibrated every 6–12 months in the lab using NOAA‐CMDL gas standards. We calculated the average daily energy balance closure, which had a slope of 0.89 and an *r* ^2^ of .92 (data not shown). All abbreviations and units for all variables used are given in Table 1.

### 2.4 Data quality control and gap filling

The 30 min EC data were quality assured and quality controlled (QA/QC) using the OzFlux standard processing protocol implemented through the OzFluxQC v2.9.4 python scripts as described by Isaac et al. (2017) to produce level 3 data. These level 3 data are available on the OzFlux data portal (http://data.ozflux.org.au/) under the Creative Commons Attribution 4.0 International License (CC BY 4.0). The OzFlux QA/QC process involves range tests, spike removal, screening for 30 min data where more than 1% of 10 Hz observations are missing from the 30 min average, linear corrections for sensor drift and calibration changes plus rejection of observations when wind originates from behind the 3D anemometer and tower. A number of corrections are also applied to the data during the QA/QC process, which include frequency attenuation, 2D coordinate rotation, conversion of sensible heat from virtual to actual flux, application of the WPL correction to account for density effects of heat and water vapour transfer on fluxes (Webb et al., 1980), and correction of soil water and soil heat flux measurements. The full suite of scripts of over 10,000 lines of code is available from GitHub (https://github.com/OzFlux/OzFluxQC) under the GNU Public License (Isaac et al., 2017).

Data gaps in the level 3 data were filled using a processing package DINGO (Dynamic INtegrated Gap filling and partitioning for OzFlux, Beringer et al., 2017) also developed in Python (https://github.com/jberinge/DINGO12). For data gaps of <2 h, DINGO fills using linear interpolation. For gaps in meteorological data >2 h, DINGO uses local Australian Bureau of Meteorology (BoM) automated weather stations and correlations with the flux dataset to find the BoM site with the best correlation. Meteorological data gaps were then filled using the BoM data. Air temperature, humidity, pressure, precipitation and wind speed were all gap filled in this way. We also compiled a precipitation (Precip) time series for the flux area using the gridded ERA5 precipitation product from for the European Centre for Medium‐Range Weather Forecasts (ECMWF, https://cds.climate.copernicus.eu/cdsapp#!/home). Solar radiant energy was gap filled using gridded satellite radiation and MODIS albedo data (MOD43B3). Soil water and soil temperature data gaps were filled using the BIOS2 land surface model of the Community Atmosphere Biosphere Land Exchange (CABLE) land surface system (Haverd, Raupauch, Briggs, Canadell, Davis, et al., 2013; Haverd, Raupauch, Briggs, Canadell, Issac, et al., 2013), which was driven by 5 km gridded meteorology from the Australian Water Availability Project (AWAP, Jones et al., 2009). The fraction of gap filled data varied from year to year due to fire damage, power and/or sensor failure and was 23% across the entire dataset except NEE data, which was 25% gap filled.

### 2.5 Flux partitioning

The DINGO package was used to partition NEE into GPP and *R*_eco_. Nocturnal NEE data were taken to be equal to *R*_eco_ and considered reliable when turbulent transport was sufficient, as defined by a threshold friction velocity (u*) (Goulden et al., 1996). The u* threshold for our site was determined using DINGO that implements the approach of Barr et al. (2013). Each half‐hourly value of NEE is checked and if the observed u* falls below the threshold, DINGO removes the NEE value. An artificial neural network (ANN) was trained to predict *R*_eco_ using surface *θ*_v_ (10 cm depth), soil temperature (*T*_soil_), *T*_air_ and the MODIS Normalized Difference Vegetation Index (NDVI, MOD13). Missing values of *R*_eco_ were gap‐filled using the predicted values of respiration from the ANN and the predicted values were then extrapolated to the daytime and GPP was calculated as NEE + *R*_eco_. Flux measurements at this site have been complemented by a number of previous studies including a carbon inventory and productivity (Chen et al., 2003), ecohydrology and water use (Cook et al., 1998; Hutley et al., 2000), phenology and tree–grass partitioning (Moore et al., 2016, 2017), aquatic carbon export (Duvert et al., 2020) and modelling (Birkel et al., 2020; Schymanski et al., 2007, 2008, 2009; Whitley et al., 2011).

### 2.6 Trend detection

Trend detection in the time series of meteorological, soil, LAI (environmental variables) and observed flux variables was undertaken using the non‐parametric Mann–Kendall test including Sen's slope method for significance testing at *p* < .05 (Hamed, 2008; Ito & Akihiko, 2012). Given the highly seasonal, summer (DJFM) dominant rainfall, meteorological and flux data were grouped as water years, defined as the period 1 July to 30 June the following year. This grouping, as opposed to calendar years, conveniently captures canopy dynamics over a complete wet season, which typically lasts from October to April the following year, with little to no rainfall occurring in the dry season months of May–August (Figure 1a). For all water years (1 July 2001 to 30 June 2019), 30 min gap‐filled data were used to calculate annual (water year) means of all meteorological, soil, LAI and fluxes variables LE, NEE, GPP and *R*_eco_. Trends in growing season length (GS) were defined as the number of days of each water year when volumetric soil water content (*θ*_v_) at 10 cm depth was >0.1 m^3^ m^−3^. LAI using MODIS has been previously shown to capture both the magnitude and seasonal range of LAI at this site and savannas across north Australia (Sea et al., 2011) and we used the 8‐day, 500 m resolution LAI and fractional absorbed photosynthetically active radiation (fPAR) products (MOD15A2, lpdaac.usgs.gov/dataset_discovery/modis). These were interpolated to provide a daily estimate of LAI and fPAR. Trends in ecosystem WUE (WUE = GPP/LE) and inherent ecosystem water use efficiency (IWUE) as defined by Beer et al. (2009) were also calculated:

(1) IWUE=GPP×VPD/LE

and radiation use efficiency (RUE) after Garbulsky et al. (2010);

(2) RUE=GPP/fPAR×PAR

To calculate absorbed PAR (APAR), daily measures of *R*_sw_ were converted to PAR using a site‐derived scaling factor of 0.47 (Kanniah et al., 2013). Units for these functional variables are given in Table 1.

**TABLE 1:** Description, abbreviation and units of all variables used, listed by meteorological, flux and ecosystem process variables

Description	Abbreviation	Unit
Atmospheric pressure	*P*_atmos_	kPa
Incoming short‐wave radiation	*R*_sw_	MJ m^−2^
Incoming long‐wave radiation	*R*_lw_	MJ m^−2^
Mean daily air temperature at instrument height	*T*_air_	℃
Mean daily maximum air temperature	*T*_max_	℃
Mean daily minimum air temperature	*T*_min_	℃
Mean daily minimum soil temperature	*T*_s_	℃
Wind speed	WS	m s^−1^
Vapour pressure deficit	VPD	kPa
Mean annual rainfall	Precip	mm
Volumetric soil moisture	*θ*_v_	m^3^ m^−3^
Growing season length	GS	day
Atmospheric CO_2_ concentration	[CO_2_]	ppm
Gross primary production	GPP	g C m^2^ year^−1^
Ecosystem respiration	*R*_eco_	g C m^2^ year^−1^
Net ecosystem exchange	NEE	g C m^2^ year^−1^
Latent heat	LE	W m^2^ year^−1^
Leaf area index	LAI	m^2^ m^−2^
Water use efficiency	WUE	g C kg^−1^ H_2_O
Inherent water use efficiency	IWUE	g C hPa kg^−1^ H_2_O
Radiation use efficiency	RUE	g C MJ^−1^ APAR

Data were grouped annually, seasonally as wet (DJFM) and dry season months (JJA) and for weeks of the year. For the trends in surface atmospheric [CO_2_], we used mean daily data from the joint CSIRO and Bureau of Meteorology's greenhouse gas monitoring station at Cape Grim, Tasmania (https://gaw.kishou.go.jp/search/station#CGO). As a carbon balance was not the focus of this study, flux variables GPP, *R*_eco_, NEE were reported as positive values.

### 2.7 Relative importance of flux drivers

A random forest (RF) learning method (Breiman, 2001) was used to rank the importance of flux drivers using correlated candidate variables *R*_sw_, *T*_max_, *T*_min_, VPD, [CO_2_] and *θ*_v_. This analysis was undertaken using monthly means to examine the seasonality of importance of each driver as RF model estimates the relative contribution each variable makes to the prediction of GPP, NEE, *R*_eco_ and LE. The RF algorithm used was from the *Scikit*‐*learn* Python module as described by Pedregosa et al. (2011).

### 2.8 Attribution of flux drivers

To compliment the trend and RF analysis, we used the mechanistic terrestrial biosphere model T&C (Fatichi et al., 2012, 2014; Fatichi & Pappas, 2017) to run a number of numerical attribution experiments in order to examine the climatological and physiological basis for trends in the fluxes. In these simulations, we ran the model with all input variables as the computed climatology's (see below) to remove the influence of any trend in climate inputs. We then undertook additional model runs, each time adding climate input variables using the observed time series to assess the relative influence of the natural interannual variability (IAV) of each of the flux drivers.

The T&C model simulates energy, water and carbon exchanges at the land surface at hourly time steps and includes essential components of the hydrological and carbon cycles. Mass and energy fluxes control the temporal dynamics of the vegetation (carbon pools) that drive land‐atmosphere exchange that is determined by the vegetation's biophysical structure and physiological properties. LAI is treated a prognostic variable which varies in response to environmental conditions and phenology. The model can be used for distributed simulations at catchment scales or applied as one‐dimensional vertical model as was implemented in this study. A detailed description of T&C’s structure and process parameterizations is presented in Fatichi et al. (2012) and it has been shown to have significant predictive capability across a range of ecosystems worldwide (e.g., Botter et al., 2021; Fatichi & Ivanov, 2014; Fatichi et al., 2012, 2014; Mastrotheodoros et al., 2017, 2020; Manoli et al., 2018; Paschalis et al., 2015).

At the Howards Springs savanna site, the model was implemented by discretizing the soil column into 26 vertical layers with increasing depth from near the surface to a maximum depth of 15 m. Two vegetation components were considered in the model; a woody overstorey dominated by *Eucalyptus* tree species, and an understorey dominated by tropical C4 grasses. Tree overstorey height was set at 18 m and seasonal C4 grass canopy height was prognostically simulated and ranged from 0 to 0.4 m. The maximum rooting depths for the two vegetation types were assumed to be 4 and 0.3 m, respectively. Model parameters describing phenology (i.e. radiant energy and soil water thresholds for leaf onset and the water stress control of leaf shedding), photosynthetic capacity (maximum Rubisco capacity) and biophysical variables (specific leaf area) were manually adjusted to values representative of the dominant site species (Eucalyptus tree species and C4 grasses) and to match observed phenological dynamics, as is common practice when implementing the T&C model (Fatichi & Pappas, 2017; Manoli et al., 2018; Mastrotheodoros et al., 2017). Nutrient dynamics were neglected in these simulations and we assumed that the vegetation exists in a mature phase in equilibrium with its nutritional environment. Fire occurrence at the site may influence nutrient dynamics; however, the fire regime has remained largely unchanged at the site over the last 30 years and given the relatively low severity and intensity of these events, fire is likely to have limited impact on nutrient dynamics. Livesley et al. (2011) examined fire effects on soil fluxes of CO_2_, CH_4_ and N_2_O at the Howard Springs site and found no impact on fluxes, suggesting negligible impact on the soil microbial community. As such, nutrient dynamics may be dominated by seasonal rainfall and inherent soil properties rather than fire at the site. For the purposes of our model experiment, the vegetation was assumed to be in nutritional equilibrium. GPP, LE and a number of other ecohydrological variables were simulated at hourly time scales to compute daily, monthly and annual means for the 18‐year data period.

### 2.9 Numerical experiment

Meteorological input variables were sourced from the gap‐filled tower observations and precipitation, which was subject to data gaps, and for model runs, we used the gridded ERA5 precipitation product from the European Centre for Medium‐Range Weather Forecasts (ECMWF, https://cds.climate.copernicus.eu/cdsapp#!/home) to replace the whole time series for consistency. Input variables used were hourly ERA5 precipitation (Precip), flux tower *T*_air_, *R*_sw_, incoming longwave radiation (*R*_lw_), RH%, wind speed, surface atmospheric pressure (*P*_atm_) and [CO_2_]. In addition to the observed meteorological variables, for each of these variables, a climatology was generated by computing the mean value for each hour of each day of the year (DOY) using the observed time‐series data. For precipitation, ERA5 data were provided at a daily timestep and hence we distributed the daily mean precipitation uniformly over 5 h (18.00–22.00) since using average precipitation every hour of each day would lead to an unrealistic continuous drizzling. The resultant climatology for each variable was used as model input data representing a scenario where input drivers are detrended with no IAV but diurnal patterns and seasonal cycles are preserved. This results in a simulation with no trend in targeted flux variables, GPP and LE.

First, we undertook a simulation where the model was forced using all observed input data providing a mean annual GPP and trend for the site over the observation period. The control simulation, forced with observed meteorological inputs, was used to compare model results with flux‐tower observations. To isolate the relative influence of a particular variable or combination of variables on the simulated GPP, the T&C model was then forced using the climatologies for each variable except for one or more ‘enabled variables’, where the observed time series was used, replacing the de‐trended and averaged climatology. By forcing the model with observations in a systematic stepwise manner, and comparing it with the control simulation, we could isolate the effect of each variable(s) on the magnitude and trend of modelled GPP. Subsequent model runs were undertaken with additional input variables enabled.

Several single model runs were also used to test the influence of Precip and its timing as detailed in Table 4 (scenarios 1–14). To examine the importance of Precip at different times of the seasonal cycle (beginning and end of the wet), we created hybrid time series for particular days of the year (DOY) where we enabled Precip climatology, and for other key periods, we used the observed precipitation (scenarios 12–14). This combination allowed evaluation of the effect on trends arising from variability in precipitation in specific periods of the seasonal cycle. We selected DOY 100–150 representing the end of wet season, beginning of the dry season, plus DOY 270–300 (end of dry season and beginning of the wet season). The T&C model was run at an hourly scale for 2001–2019 and the simulated hourly GPP and LE was aggregated to annual totals for the ecosystem as well as tree and grass components per water year. As with the observations, the Mann–Kendall test and Sen's slope statistic were used with the model data to detect significant trends (*p* < .05).

## 3 RESULTS

### 3.1 Site characteristics

A time series of site meteorology and *θ*_v_ is shown in Figure 1, represented as weekly ensemble means for the observation period. MAP calculated for all water years using the gridded ERA5 data was 1608 ± 403 mm, with a highly seasonal distribution (Figure 1a). For the same period, Darwin Airport recorded an annual mean of 1765 ± 461 mm (Bureau of Meteorology, Station 14015, 30 km NW of the flux site). Seasonal variation of *R*_sw_ and *T*_air_ was small. Mean daily *R*_sw_ ranged from 17 to 22 MJ day^−1^ (Figure 1b) and daily mean *T*_max_ ranged from 30 to 34°C, although *T*_min_ varied seasonally by ~8°C (Figure 1c). VPD and volumetric soil water (*θ*_v_) were highly seasonal (Figure 1c,d). Surface (0.1 m depth) and deep *θ*_v_ (1 m) were closely coupled (Figure 1d), and both soil horizons had a similar seasonal range of 0.1 m^3^ m^−3^.

### 3.2 Trend detection

**FIGURE 2:**
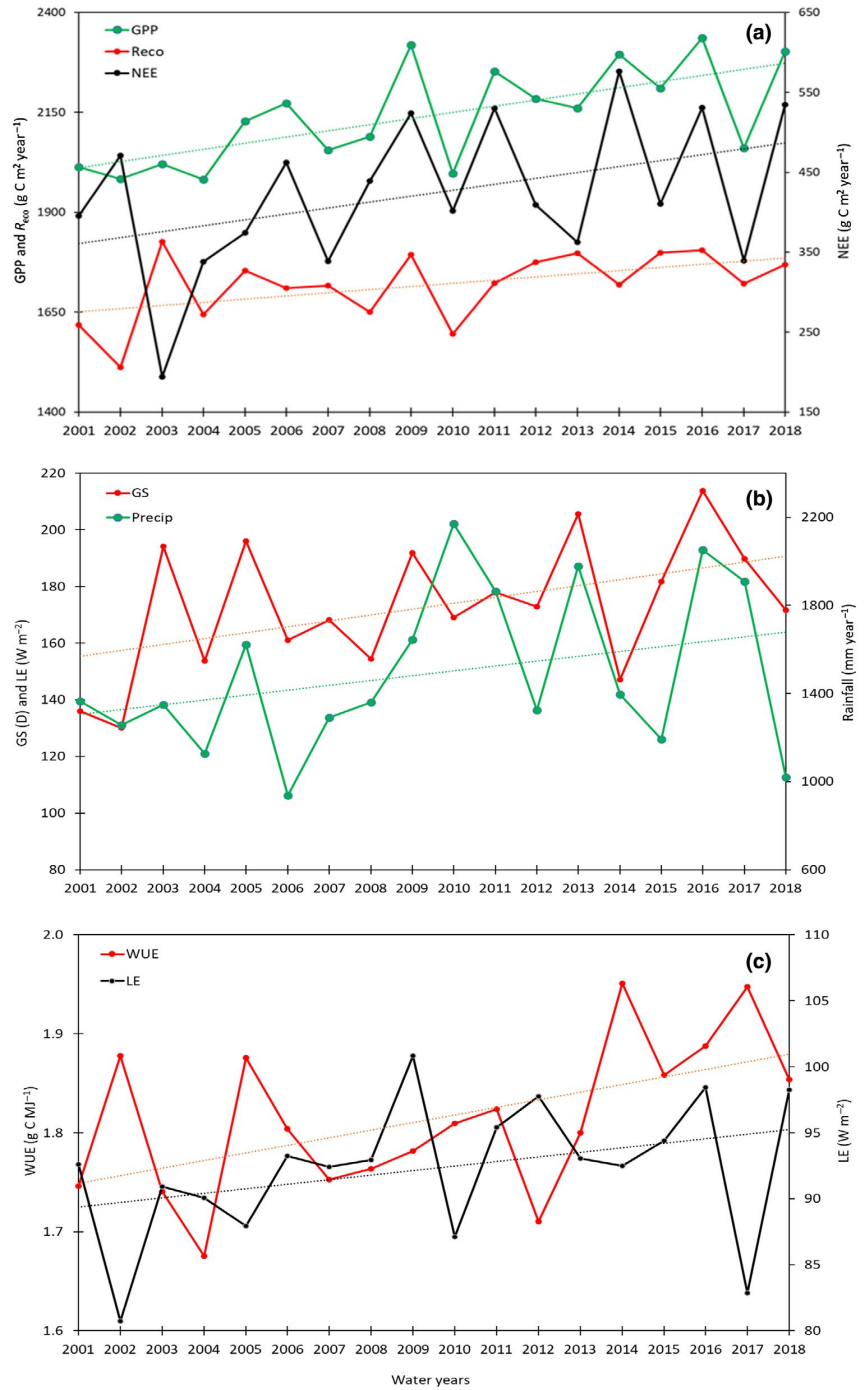
Annual time series for statically significant flux variables (*p* < .05) with linear trend lines shown. Mean annual values were calculated using data aggregated as water years (30 June to 1 July) for (a) gross primary productivity (GPP), *R*_eco_ and net ecosystem exchange (NEE), (b) growing season length (GS) and Precip, and (c) latent energy (LE) and ecosystem water use efficiency (WUE)

Significant slopes are given in Tables 2 and 3 for environmental and flux variables, respectively, and plotted in Figure 2 with trend lines. Significant slope estimates for functional properties WUE, IWUE and RUE are also given in Table 3.

**TABLE 2:** Trend analysis for meteorological variables calculated using annual means and seasonal monthly means (wet season = DJFM, dry season = JJAS) for the 18 water years

Period	*R*_sw_ (MJ m^−2^ year^−1^)	*T*_air_ (℃ year^−1^)	VPD (kPa year^−1^)	LAI (m^2^ m^−2^ year^−1^)	Precip (mm year^−1^)	*θ*_v_ (m^3^ m^−3^ year^−1^)	GS (day year^−1^)	[CO_2_] (ppm year^−1^)
Annual	NS	NS	NS	NS	NS	NS	2.4 (.05)	2.11 (<.001)
Wet	NS	NS	NS	NS	NS	NS	NS	
Dry	NS	0.11 (<.001)	NS	NS	NS	NS	NS	

Note

The non‐parametric Mann–Kendell test and Sen's slope estimator was used to identify significant trends. For significant trends, *p*‐values are given in parentheses. Growing season length (GS) was defined as the number of days when *θ*_v_ was >0.1 m^3^ m^−3^.

Abbreviations: LAI, leaf area index; NS, not significant; VPD, vapour pressure deficit.

Observed mean annual GPP, NEE and *R*_eco_ over the observation period were 2144 ± 123, 424 ± 95 and 1718 ± 85 g C m^−2^ year^−1^, respectively, with annual trends of 15.4, 7.4 and 8.0 g C m^−2^ year^−2^ for these same variables. All were significant at *p* < .05 (Table 3). Observed mean annual LE was 92.3 ± 5.2 W m^−2^ and WUE 1.81 ± 0.1 g C kg^−1^. Trends in GPP, NEE and *R*_eco_ were larger during the dry season months and were 26.7, 15.4 and 11.3 g C m^−2^ year^−2^, respectively (Table 3). There was a weak but non‐significant trend in LE of 0.34 W m^−2^ year^−1^ (*p* < .12), equivalent to 4.4 mm year^−1^. The annual linear trend in GPP was 0.72% per year relative to the mean, more than double the increase in LE (0.37% per year). Given this result, there were significant trends in annual and dry season WUE and dry season RUE and IWUE. Trends in annual growing season length (GS, 2.4 days year^−1^) and [CO_2_] (2.11 ppm year^−1^) were also significant, as was *T*_air_ during the dry season months (0.11°C year^−1^). No trends were detected during the wet season for any meteorological or flux variable (Tables 2 and 3).

**TABLE 3:** Trend analysis for flux and ecosystem functional variables calculated for water years using annual and seasonal monthly means (wet season = DJFM, dry season = JJAS) for the 18 data years

Period	GPP	LE	NEE	*R*_eco_	WUE	IWUE	RUE
Annual	15.4 (<.001)	0.34 (.12)	7.4 (.07)	8.0 (.04)	0.0077 (.02)	NS	NS
Wet	NS	NS	NS	NS	NS	NS	NS
Dry	26.7 (<.001)	NS	15.4 (.02)	11.3(.02)	0.016 (<.01)	29.7 (<.0)	0.016 (<.0)

Note

A 60‐day exclusion period was used to mask data collected during post‐fire events. For significant trends, *p*‐values are given in parentheses. Units for trends in GPP, NEE and *R*_eco_ are g C m^−2^ year^−2^, WUE is g C kg^−1^ year^−1^, LE is W m^−2^ year^−2^ and RUE g C m^−2^ MJ^−1^ APAR year^−1^.

Abbreviations: GPP, gross primary productivity; IWUE, inherent water use efficiency; LE, latent energy; NEE, net ecosystem exchange; NS, not significant; RUE, radiation use efficiency; WUE, water use efficiency.

**FIGURE 3:**
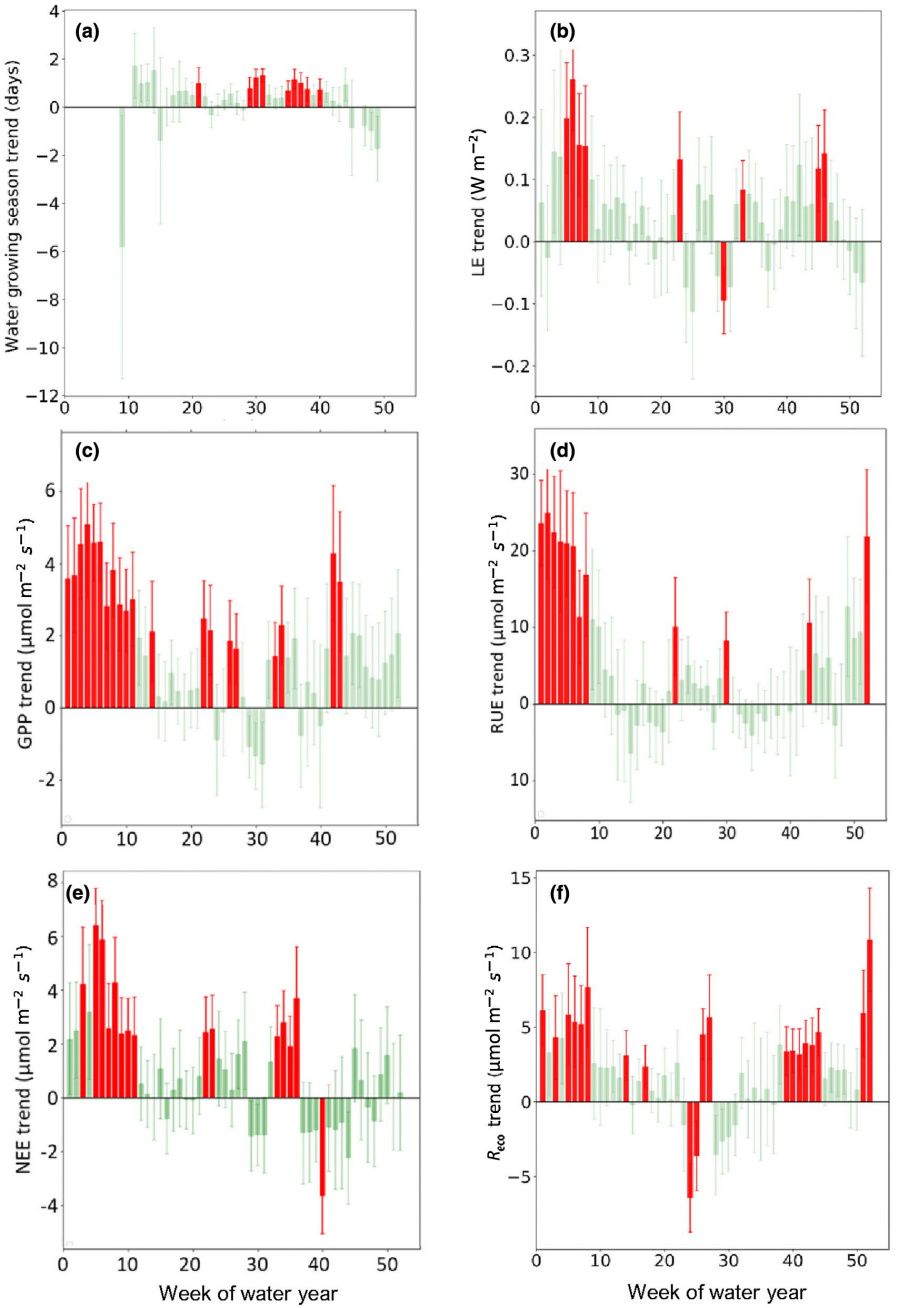
Temporal distribution of observed trends for variables with statically significant slopes (*p* < .05), with data grouped by week of water year. Ensemble mean trend (±standard deviation [SD]) for each week of all water years (*n* = 18) are plotted by week. Weeks with a statically significant trend (*p* < .05) are coloured red for trends in (a) the change in growing season (GS), the number of days per week with surface soil moisture greater than 0.1 m^3^ m^−3^, (b) latent energy (LE), (c) gross primary productivity (GPP), (d) radiation use efficiency (RUE), (e) net ecosystem exchange (NEE) and (f) *R*_eco_

To further examine the timing of trends seasonally, they were calculated for each week of the year using the 18‐year record (Figure 3). Figure 3a shows a significant (*p* < .05) increasing wet season growing season length (GS), indicating a wetting tendency and potentially a lengthening of the monsoon season given LE was increasing. Trends in LE, GPP, NEE, RUE and *R*_eco_ occurred predominantly during the dry season (Figure 3b–f, July–August, weeks 1–15 of the water year) and during the transition from the wet to dry season (weeks 41–48). Trends in GPP were significant for all weeks of the dry season, late dry season and into October, the wet to dry transition (weeks 1–15, Figure 3c). Trends in *R*_eco_ were evident at the start and end of the monsoon as *R*_eco_ is highly sensitive to soil water in this ecosystem (Figure 3f).

### 3.3 Relative importance of flux drivers

**FIGURE 4:**
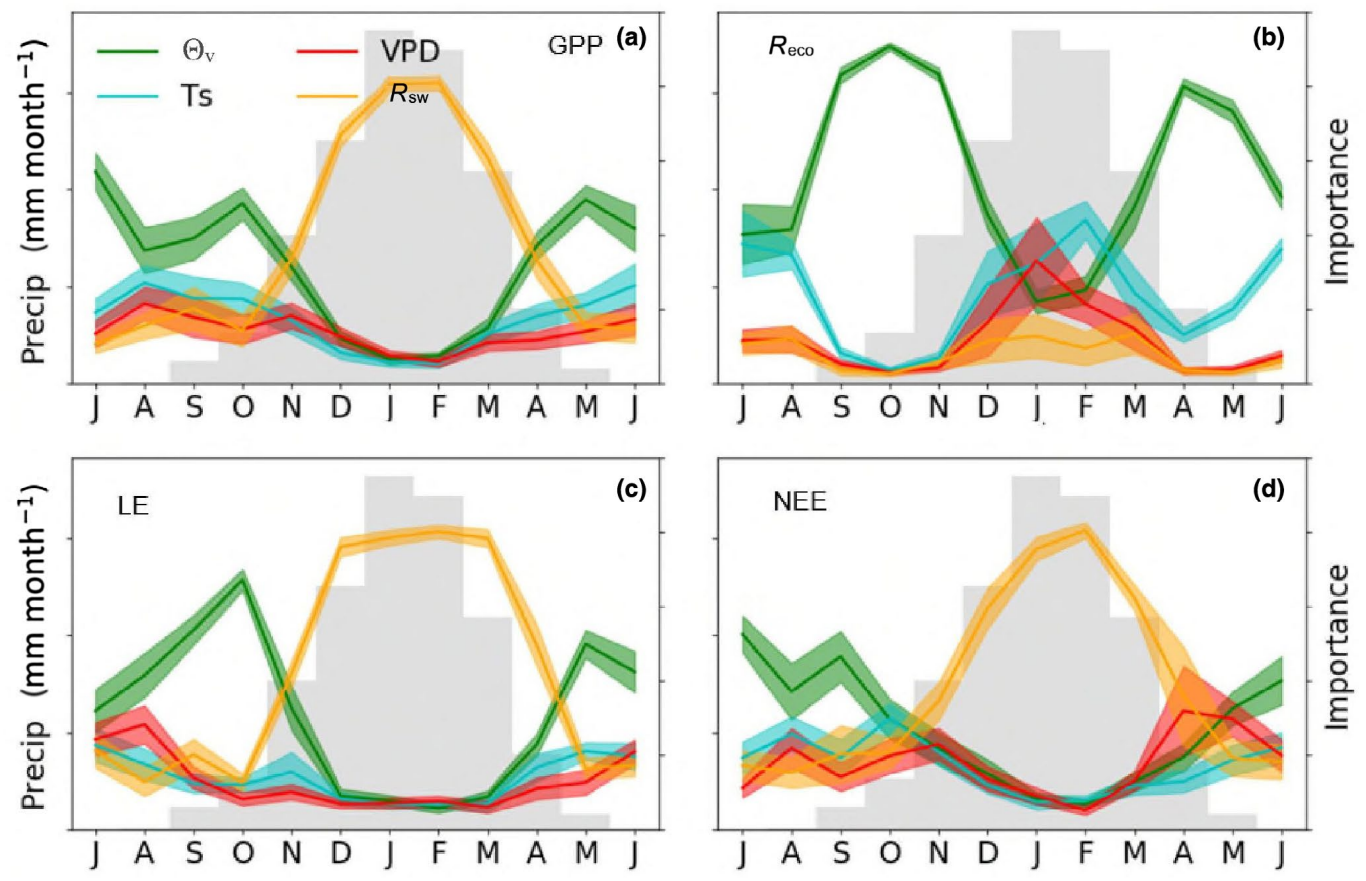
Seasonal patterns of importance values from Random Forest analysis. Daily data were binned into months and run independently using *θ*_v_, vapour pressure deficit (VPD), *T*_s_ and *R*_sw_ as predictive variables. Solid coloured lines are the monthly mean importance values (RH axis) using these predictors of (a) gross primary productivity (GPP), (b) ecosystem respiration (*R*_eco_), (c) latent energy (LE) and (d) net ecosystem exchange (NEE). Shading is ±1 standard deviation of mean importance. Grey shaded bars are mean monthly Precip (LH axis)

The RF modelling showed that the importance of flux drivers differed with season (Figure 4). For GPP, NEE and LE, *R*_sw_ dominated during the wet season and *θ*_v_ during the dry season. During transitional periods, from wet to dry and dry to wet seasons, these two variables were co‐dominant. Drivers for *R*_eco_ differed with *T*_soil_ of high importance during the dry season, whereas *θ*_v_ was dominant during the seasonal transitions (May–April and October–December, Figure 4b). VPD was only important as a driver of *R*_eco_, occurring during the peak of the wet season (January) when mean *θ*_v_ is approaching maximal values. Lastly, we wanted to see whether the importance of these drivers had changed over the 18 years, so we applied our trend analysis to the annual importance values of each driver for the fluxes (data not shown). However, there were no statistically significant trends indicating that the importance of those drivers had changed over time.

### 3.4 Model attribution experiment

**FIGURE 5:**
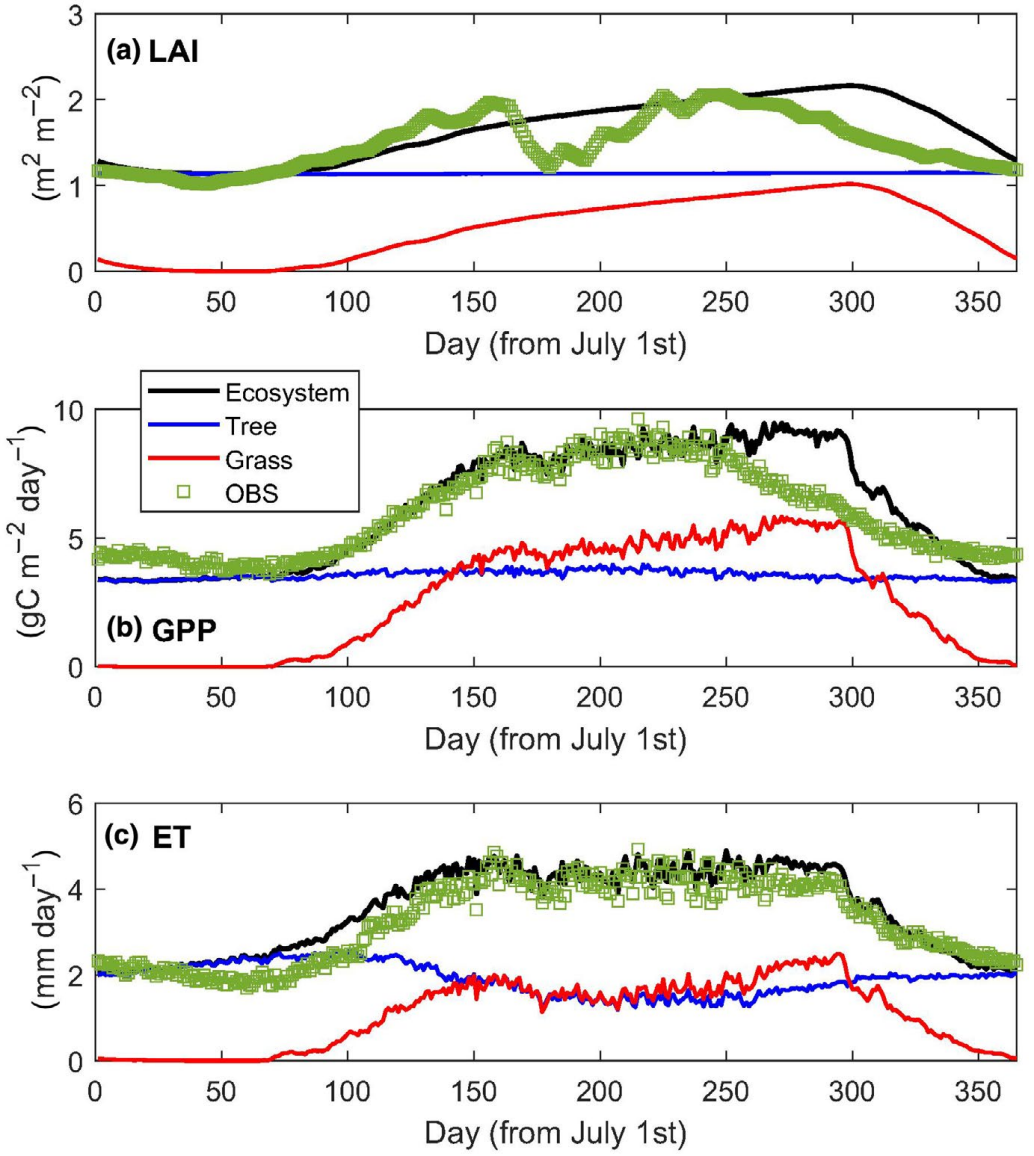
Time series (day of year, DOY) of the T&C model output data for daily mean (a) leaf area index (LAI), (b) gross primary productivity (GPP) and (c) latent energy (LE) with fluxes partitioned for grass, tree and ecosystem. Observed GPP, LE and smoothed MODIS‐derived LAI for the site are also given

The RF and trend analysis of environmental drivers of fluxes are correlative only, identifying drivers of variability, but this provides some understanding of the underlying processes. To complement these analyses, we used the mechanistic T&C model to undertake an attribution experiment to examine the climatological and physiological basis for the observed trends in the flux data. For this purpose, we focused on mean values and trends in GPP, LE and WUE of tree and grass components and the ecosystem as a whole. Model outputs for all scenarios for GPP are given in Table 4. The T&C model predicted hourly GPP and LE with an *r* ^2^ of .80 and .92, respectively, and seasonal dynamics were well captured (Figure 5), except for transitional months (April–May) when LAI and GPP were overestimated (Figure 5a,b). Similarly, observed and modelled mean annual LE (1283 vs. 1198 mm year^−1^) and WUE (1.79 vs. 1.77 g C kg^−1^ year^−1^) were in good agreement. At an annual time‐scale, simulated GPP of 2271 ± 986 g C m^−2^ year^−1^ was close to the observed mean annual GPP of 2144 g C m^−2^ year^−1^ (Table 4).

**TABLE 4:** Attribution of experimental outputs from the T&C model for mean annual GPP and trends, partitioned into overstorey tree canopy (C3, evergreen) and the grass‐dominated understorey (C4, annual) based on the seasonal dynamics of simulated LAI for trees and grasses

Scenario ID	Variables enabled	GPP mean (g C m^−2^ year^−1^)			GPP trend (g C m^−2^ year^−2^)		
		Ecosystem	Tree	Grass	Ecosystem	Tree	Grass
1	All	2271	1294	976	29.4	13.4	15.9
2	*P*_atmos_	2819	1487	1331	0.60	0.12	0.48
3	*P*_atmos_ + *R*_lw_	2811	1487	1324	0.84	0.10	0.73
4	*P*_atmos_ + *R*_lw_ + [CO_2_]	2813	1488	1325	10.2	8.59	1.62
5	*P*_atmos_ + *R*_lw_ + [CO_2_] + RH% + wind speed	2809	1487	1327	11.7	10.6	1.07
6	*P*_atmos_ + *R*_lw_ + [CO_2_] + RH% + wind speed + *R*_sw_	2586	1338	1248	13.6	11.5	2.06
7	*P*_atmos_ *R*_lw_ + [CO_2_] + RH% + wind speed + *R*_sw_ + *T*_air_	2548	1295	1253	18.2	13.3	4.83
8	Precip	2532	1478	1054	13.3	0.04	13.3
9	Precip + *T*_air_	2463	1430	1032	17.0	3.86	13.1
10	Precip + [CO_2_]	2533	1479	1053	22.9	8.58	14.3
11	Precip + *T*_air_ + [CO_2_]	2464	1432	1032	26.5	12.2	14.2
12	Precip (only DOY 100–150 and 270–300)	2595	1472	1122	7.1	−0.21	7.32
13	Precip (only DOY 100–150)	2749	1483	1266	3.0	0.07	3.01
14	Precip (only DOY 270–300)	2659	1476	1183	4.9	−0.22	5.13

Note

The model was forced using the detrended climatologies calculated for each variable except for those listed as an enabled variable(s) which were the observed time series.

Abbreviations: DOY, day of the year; GPP, gross primary productivity; LAI, leaf area index.

In the baseline simulation, all climate input variables were the observed series in the model. The simulated annual ecosystem GPP showed a significant and increasing trend with a slope of 29.4 g C m^−2^ year^−2^ (scenario 1 trend, Table 4), double the observed annual GPP trend of 15.4 g C m^−2^ year^−2^ (Table 3). The sequential scenario runs, where one variable was changed at a time, enabled quantification of the relative contribution of each variable with respect to the baseline trend (scenario 1). The ecosystem trend was partitioned equally between tree (13.4 g C m^−2^ year^−2^) and grass (15.9 g C m^−2^ year^−2^) components. At the ecosystem scale, enabling [CO_2_] (scenario 4) accounted for 35% of the simulated baseline and Precip alone (scenario 8) accounted for 45%. Including *T*_air_ (scenario 9) plus [CO_2_] and Precip (scenario 11) resulted in a simulated trend of 26.5 g C m^−2^ year^−2^, which is 90% of the modelled baseline trend.

Modelled tree GPP showed a strong response to [CO_2_] and this variable alone accounted for 63% of the tree response, with *T*_air_ contributing an additional 29% (scenario 11, tree trend). Together, *T*_air_ and CO_2_ accounted for 93% of trend in tree GPP with Precip having a limited effect (scenarios 8–11), and no response during seasonal transition periods (scenarios 12–14). By contrast, the trend in grass GPP of 15.9 g Cm^−2^ year^−2^ was largely accounted for by Precip alone (84%, scenario 8 grass, Table 4), and the inclusion of [CO_2_] only contributed an additional 6% to the simulated understorey trend (scenario 10). There was a sensitivity to Precip during transitional periods (scenario 12), the early growing season, pre‐monsoonal month of September (scenario 14), which contributed 32% to the overall grass trend.

Trends in modelled ecosystem WUE were significant and positive. However, like the modelled fluxes, trends in ecosystem WUE were double (0.0154 g C kg^−1^) the observed ecosystem WUE trend (0.0078 g C kg^−1^). As with the modelled GPP trend, the trend in ecosystem WUE was reproduced in scenario 11 (Precip, [CO_2_] and *T*_air_ enabled) and resulted in an ecosystem WUE trend of 0.0168 g C kg^−1^ of which the trend in tree WUE accounted for 83%.

## 4 DISCUSSION

### 4.1 Trends in observed fluxes and drivers

Observations from this site indicated significant positive annual trends in GPP, NEE, LE, *R*_eco_, WUE and RUE. An adequate period of observation is required to detect trends in fluxes over and above natural variability and systematic measurement errors. Baldocchi et al. (2018) estimated the minimum detectable trend in carbon flux measurements as a function of observation period using an assumed systematic error inherent in EC systems of 30 g C m^−2^ year^−1^ (after Elbers et al., 2011). Based on their analysis, an 18‐year flux record can detect a trend in flux of ~3 g C m^−2^ year^−2^, which suggests the trends observed for GPP, *R*_eco_ and NEE (Table 3) are likely to be robust.

There are few long‐term (>10 years) flux studies in savannas and most have been undertaken in semi‐arid climate zones. Archibald et al. (2009) reported on the dominant drivers and magnitude of interannual variability of GPP, NEE and *R*_eco_ at a semi‐arid savanna site in Kruger National Park. A 5‐year flux record at the Kruger Park site was used to train an artificial neural network model which was combined with that site's 25‐year record of site meteorological and phenological data to assess interannual variability of GPP, NEE and *R*_eco_. While trend analysis was not feasible, the study demonstrated the importance of the timing and magnitude of the sporadic rainfall events on NEE, which was then influenced by radiant energy load, growing season length and soil water dynamics. Ma, Baldocchi, et al. (2016) reported carbon fluxes from Mediterranean oak‐savanna and grassland sites in southern California for a similar period as this study (2000–2015) and they reported an increase in growing season length, but non‐significant and negative trends in GPP, NEE and *R*_eco_. As with the semi‐arid Kruger savanna site (Archibald et al., 2009), the interannual variability of C fluxes was closely coupled to rainfall variability and growing season length. Rainfall and temperature extremes from previous years influenced variation in annual carbon balance of the following year, a slow‐acting ‘legacy’ response (Ma, Baldocchi, et al., 2016).

The Howard Springs site differs from these drier savannas with a higher GPP and LE that is more similar to reported fluxes for seasonally dry and wet forests (Chen et al., 2017) with strong trends in GPP, NEE, LE, *R*_eco_, WUE and RUE. Significant increases in GPP and NEE largely occurred in the dry season (Table 3, Figure 3) and thus were derived from the C3 woody components of the ecosystem. Tree growth occurs in the wet season, but persists into the dry season months through to June–July (Chen et al., 2003), whereas the growing season of the grass dominated understorey is restricted to the wet season, although grass production does increase following large wet seasons (Yates et al., 2020).

While a change in annual rainfall was not significant during our observation period, of the 18 water years, nine received above‐average rainfall that ranged from 185 to 1237 mm above the 1720 mm average. Wet year rainfall anomalies were on average 100 mm larger than dry year anomalies, and the site rainfall IAV may have masked statistically significant trends that may only be revealed by longer time series. Precipitation data from Darwin Airport Bureau of Meteorology station (~29 km west of the site) showed a significant 4.5 mm year^−1^ trend from 1941 to 2018 (*p* < .02). In addition, our soil–water‐derived proxy for growing season length (GS, number of days when *θ*_v_ > 0.1 m^3^ m^−3^) showed a significant positive trend in the wet seasons, which suggests a wetting ‘tendency’ at the site. Such a trend would potentially benefit both grass and tree components as roots of both lifeforms occupy the upper 50 cm of soil during the wet season. Root excavations (Eamus et al., 2002) and root productivity studies (Chen et al., 2004; Janos et al., 2008) demonstrated that both tree and grass roots are largely distributed in the upper 1 m of soil, with 70%–90% of the root biomass occurring on the top 0.5 m. This is consistent with the T&C model predictions of increasing trends in both tree and grass GPP (Table 4) but in reality, this would be restricted to the wet season only.

The prominent seasonal dry season trend in fluxes we observed may relate to slow‐acting legacy effects as described by Ma, Baldocchi et al. (2016). Soil moisture dynamics at the surface (10 cm) are closely coupled with the soils at depth (1 m, Figure 1d) due to the well‐drained, coarse‐textured soil at the site and drainage to deep soil horizons is a significant component of the site water balance. High intensity monsoonal rainfalls, especially during above‐average wet seasons, results in a drainage excess that recharges sub‐soils (Cook et al., 1998). Soil water data at depth are only available from October 2009 to July 2019. Weekly mean *θ*_v_ at 1 m shows a significant trend of 0.63% per year (*p *< .04) and years that develop significant drainage to the sub‐soil (1–5 m) provides a critical moisture reserve that maintains the gas exchange of the evergreen *Eucalyptus*/*Corymbia* tree species through the dry season (Figure 5b,c), as has been observed by previous seasonal measures of tree transpiration (Hutley et al., 2000; O'Grady et al., 1999).

The strong positive GPP trend we observed in the dry season (Figure 3c) is consistent with a longer growing season and a response to elevated [CO_2_] from the C3‐dominated tree canopy as there is no active C4 grass growth during the dry season. Any increase in dry season GPP and subsequent growth is likely to be accompanied by an increase in *R*_eco_ and there was a significant dry season trend of 11.4 g m^−2^ year^−2^ (Table 3) equivalent 1% per year, although this trend was 50% that of dry season GPP and 25% of NEE. Partitioning of *R*_eco_ into heterotrophic and autotrophic respiration was not undertaken, but there may also be a response to increased dry season *T*_air_ which also showed a significant dry season trend (Table 2). A positive trend in GPP and NEE could be related to woody thickening or encroachment that is being observed across savannas and rangelands, defined as increasing density of trees and woody shrubs (Stevens et al., 2016). This process is typically driven by changes in land use and fire suppression, but CO_2_ fertilization effects are also likely to drive increases in GPP, especially the C3 woody vegetation over the C4 grasses (Bond & Midgley, 2012; Cowley et al., 2014; Scheiter et al., 2015), altering savanna structure and function.

**FIGURE 6:**
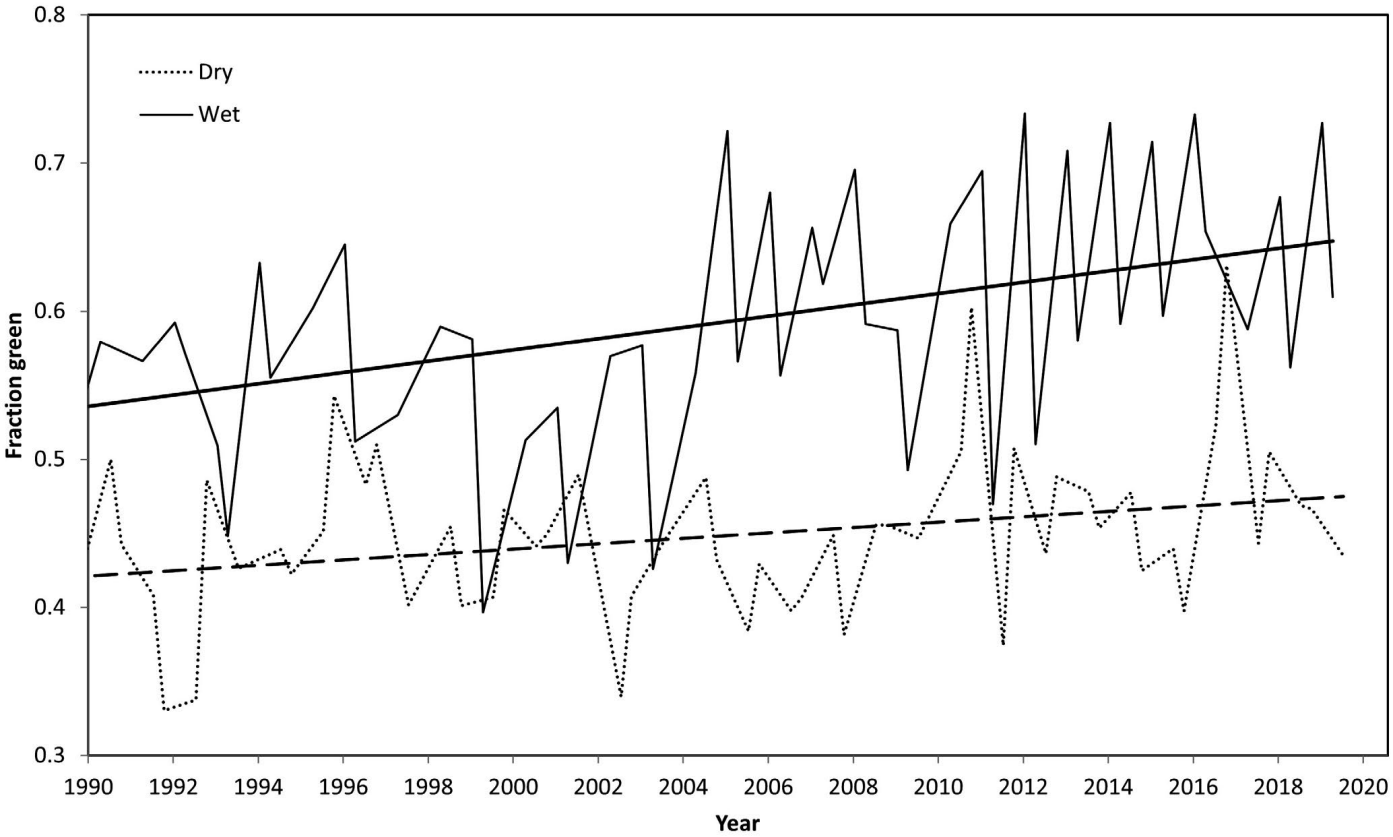
Long‐term time series of quarterly fractional green cover partitioned into wet and dry seasons Cover data were generated using the VegMachine online tool that estimates fractional green cover for a user‐selected polygon (flux footprint) from 30 m Landsat imagery that is calibrated for Australian savanna and rangelands (Beutel et al., 2019)

Fire frequency at the Howard Springs site is high (two in 3 years) yet site inventory studies at long‐term plots within the flux footprint show that woody biomass is increasing at the site (Chen et al., 2003), consistent with our observed trends in fluxes. More recent stem increment measurements over an 8‐year interval (2008–2015) estimated the tree biomass increase at 45 g C m^2^ year, a growth rate of ~1.3% per year relative to the standing biomass (M. Rudge, L. B. Hutley, J. Beringer, unpublished data). These data show tree biomass, and not stem density, increasing at Howard Springs, suggesting a physiological response of existing individual trees and shrubs. While these changes in growth are not reflected in MODIS‐derived LAI (Table 2), using a longer time series and the higher‐resolution Landsat imagery, quarterly fractional green cover shows significant trends across the fetch area in the wet (*p* < .01) and dry seasons (*p* < .055) and annually (*p* < .015) at 0.32% per year (Figure 6). These data were generated for the site's flux footprint using the online tool VegMachine (Beutel et al., 2019, https://vegmachine.net/) which estimates fractional green cover using 30 m Landsat imagery calibrated for Australian savanna and rangelands (Lehmann et al., 2013).

The GPP and NEE trends may also reflect a longer‐term ecosystem trajectory from cyclone disturbance as the Howard Springs site is within 25 km of NT coastline and experiences major cyclonic disturbances on a ~50‐year cycle, such as the two major cyclones over the last 100 years (1896 and 1974), whose effects are evident in stand structure via distinct recruitment events following the cyclones (Hutley & Beringer, 2011). This highlights the importance of interpreting flux measures in short‐, medium‐ and long‐term contexts (Hutley et al., 2013).

### 4.2 Attribution of trends in GPP

The T&C model captured the magnitude of annual fluxes well and like the observations, predicted strong trends in GPP and WUE, with trees and grasses contributing equally to this trend (Table 4). This finding differed from the observations, where the ecosystem‐scale GPP trend originated from a strong trend in the dry season (Table 3) with no trends evident in the wet season, suggesting a strong tree response. Of note was the strong modelled tree response to CO_2_ and *T*_air_ (scenario 11, Table 4), environmental drivers that both showed significant trends, although *T*_air_ was only significant in the dry season. From 2001 to 2018, [CO_2_] increased by ~38 ppm, a sufficient increase to drive an increase in C3 photosynthesis (Walker et al., 2021).

Despite frequent fire at the site, which consumes and releases sequestered carbon (Beringer et al., 2007), observations and modelling of GPP and WUE, plus site‐specific measures of canopy cover change and tree increment increases all suggest an increase in sequestration with a CO_2_ fertilization effect evident. Vegetation of the understorey in the T&C model consisted exclusively of C4 grass and the understorey trend in GPP was largely attributed to precipitation, not [CO_2_], as would be expected from a C4 dominated canopy and is consistent with a wetting tendency as evidenced by the trend of growing season length. The large overestimate in modelled trends in GPP and WUE relative to observed trends may relate to the fact that the savanna fires (two in every 3 years) are not accounted for in the model. While fire only occurs in the dry season and does not directly affect grass GPP, woody canopy elements are scorched, damaged and consumed by fire; especially severe fires that can release 50% of annual NEE (Beringer et al., 2007). Fire results in carbon loss from the ecosystem that constrains productivity, especially woody productivity, but this process was not considered by the model as our goal here was to examine the likelihood of observed trends being driven by changes in environmental drivers. In effect, trends predicted by the T&C model can be considered as a ‘fire‐free’ potential productivity and WUE for this site.

Given the long history of flux data availability from the Howard Springs site, it has been used in several other model experiments (Whitley et al., 2017). Schymanski et al. (2015) used a physiologically based vegetation optimality model (VOM) to simulate trends in GPP, LE and WUE at Howard Springs. VOM assumes vegetation is optimally adapted to environmental conditions such that root, canopy and water use dynamics maximize the long‐term ‘net carbon profit’ (NCP). Schymanski et al. (2015) forced VOM using a 35‐year site climatology and ran simulations at differing but constant levels of [CO_2_], namely 317, 350 and 380 ppm, which were representative of [CO_2_] in 1960, 1990 and 2005, respectively. For Howard Springs, when simulated GPP from trees and grasses was combined, GPP increased by 26% at 380 ppm relative to 317 ppm, implying a 1.3‐fold sensitivity of GPP to increasing [CO_2_]. In addition, the VOM simulations predicted a 1.6% trend in tree cover for a 1% trend in [CO_2_] at Howard Springs (Schymanski et al., 2015; table 6), which is similar to the observed increase in stem biomass increment of 1.3% per year. In the VOM model, this tree increment was accompanied by an increase in tree rooting depth and simulations suggested that the observed GPP trend is likely to be caused by increasing [CO_2_] alone, not necessarily by increasing rainfall, consistent with outcomes from the T&C‐based attribution experiment for woody vegetation at the site. However, as opposed to the T&C model, the VOM predicted only a small increase in grass GPP in response to increasing [CO_2_], 3% as opposed to the 26% for trees. This is more consistent with the observations presented here, where the significant GPP trends were limited to the tree‐dominated dry season. More generally, Schymanski et al. (2015) formulated a mechanism for the effects of increasing [CO_2_], whereby an initial increase in GPP and decrease in transpiration could result in increased soil water availability, followed by increased tree cover and rooting depth in the long term, essentially inducing a higher fraction of water use and GPP from grasses to trees in the long term and potentially reverting from an initial reduction in transpiration to an increase in the long term. The observations presented here confirm a shift of GPP towards trees, with a lower, non‐significant trend found for LE.

### 4.3 Trends in GPP and WUE

Trends in GPP at Howard Springs are high when compared to other ecosystems, being nearly double the range reported for temperate forests (2–10 g C m^−2^ year^−2^, Yue et al., 2016; Zhou et al., 2017) and closer to GPP trends modelled for tropical forests of 10–20 g C m^−2^ year^−2^ when both climate and CO_2_ drivers are implemented (Ichii et al., 2005; Wang et al., 2021). It is apparent that there is an emerging difference between the observation‐based data that shows smaller trends than process‐based models who report large GPP variability and significant trends (Anav et al., 2015). By contrast, Zhang et al. (2019) simulated relatively modest increases in global GPP for 2000–2015. The trend in vegetation cover was three times that of GPP and tropical forest GPP declined by 2% per year, driven by increasing VPD, drought and land use change. Simulated trends of GPP in savanna regions were positive, contributing 33% to the global GPP trend, with croplands 51% (Zhang et al., 2019). While there is convergence in global estimates of GPP, the spatiotemporal dynamics of GPP remains poorly defined and site‐ to regional‐scale studies have a significant role to play (Ciais et al., 2020). Walker et al. (2021) reviewed current theory and multidisciplinary evidence for the effects of increasing [CO_2_] on the terrestrial carbon cycle, including fertilization effects on GPP, LE and WUE with increasing [CO_2_] responsible for about half of the global increase in photosynthesis. They used a standardized CO_2_ response ratio β (after Friedlingstein et al., 1995), defined as the ratio of a response variable (e.g. WUE) at low and high [CO_2_]. A value of 1 indicates a direct proportionality between a variable's CO_2_ response and the change in [CO_2_]. Flux tower‐derived estimates of β for GPP, at a global scale, ranged from −0.39 to 1.6 (Walker et al., 2021), highlighting the range of reported responses of GPP to CO_2_. The value of β for GPP at Howard Springs was 1.30, at the upper end of the range compiled by Walker et al. (2021), also supporting our contention that there is a CO_2_ fertilization effect evident at this site. For WUE, our value of β was lower at 0.76.

With a strong trend in GPP and a weak trend in LE, WUE increased at Howard Springs, a result consistent with observed stand‐scale predictions and global modelling of WUE (Cheng et al., 2017; Donohue et al., 2013; Keenan et al., 2013; Mastrotheodoros et al., 2017; Walker et al., 2021; Zhang et al., 2016). Savanna WUE tends to be relatively low when benchmarked across biomes. Cheng et al. (2017) predicted spatial and temporal trends (1982–2011) of WUE by upscaling leaf WUE estimates to ecosystem scales using a model that was constrained by precipitation, streamflow and FluxNet data. Tropical and temperate forests range from 3 to 4 g C m^−2^ mm^−1^ with savanna ranging from 0.5 to 2.5. For mesic savanna (>1000 mm annual precipitation), Cheng et al. (2017) reported values of ~2 g C m^−2^ mm^−1^ H_2_O, again consistent with our observed WUE of 1.8 ± 0.1 g C m^−2^ mm^−1^ and the T&C modelled WUE of 1.77 g C m^−2^ mm^−1^. Cheng et al. (2017) also predicted global trends in savanna WUE, which ranged from 0.008 to 0.010 mg C kg^−1^ year^−1^ H_2_O, also consistent with our significant annual WUE trend of 0.0077 mg C kg^−1^ year^−1^ (Table 3).

### 4.4 Conclusions

We observed significant positive trends in carbon fluxes over an 18‐year period largely driven by increasing [CO_2_]. There were clear increases in dry season GPP, NEE and *R*_eco_, with a weaker trend in LE. The magnitude of these trends resulted in an increase in ecosystem‐scale WUE, consistent with leaf‐ to ecosystem‐scale responses to elevated [CO_2_]. The site is representative of the extensive coastal, high rainfall savanna woodlands and open forests of north Australia, vegetation that shares characteristics with tropical dry deciduous forest in terms of biomass storage and productivity. These tropical systems occupy some 30 million km^2^ and make a disproportionally large contribution to the trend and interannual variability of the global carbon sink (Ahlström et al., 2015). Globally, savannas provide significant ecosystem services including food security, biodiversity and carbon sequestration, but this may be offset by land‐cover changes and more severe fires due to climate change, processes that could release significant amounts of CO_2_ to the atmosphere. Persistence of this sink in a mesic savanna of Australia is uncertain as it may eventually saturate given nutrient limitations, but thickening is likely to persist for several decades (Scheiter et al., 2015). Regional‐scale model outputs from CMIP6 of the IPCC’s AR6 suggest a small increase in precipitation (Iturbide et al., 2020) as is currently being observed across north‐west Australia. This is in contrast with the savanna and dry forest regions of southern and central America and southern Africa. Using a standardized precipitation index (SPII‐6), median precipitation is predicted to decrease by 20%–50% for southern Africa and between 30 and 70% for south and central America over the next two decades assuming an SSP5 8.5 scenario (Iturbide et al., 2020). While CO_2_ fertilization may offset impacts of declining precipitation, changes to extremes of temperature, evaporative demand and fire climatology may have significant effects on sequestration rate and stability of carbon pools in these extensive tropical ecosystems. There is a high degree of uncertainty of these outcomes, and this underpins the need for the maintenance of long‐term monitoring sites, which is crucial to infer temporal trends across these ecosystems. This becomes more imperative as climate variability increases and determining responses and detecting trends over and above IAV becomes more difficult.

## 5 CONFLICT OF INTEREST

The authors declare no conflict of interest.

## Data Availability

The data that support the findings of this study are openly available and all processed flux and site meteorology data are available at the TERN OzFlux Data Portal (https://data.ozflux.org.au/portal/home). The Howard Springs site is a registered FluxNet AU‐How. Site data are at https://data.ozflux.org.au/portal/pub/viewColDetails.jspx?collection.id=1882710&collection.owner.id=304&viewType=anonymous.
